# Pearson Correlation-Based Feature Selection for Document Classification Using Balanced Training

**DOI:** 10.3390/s20236793

**Published:** 2020-11-27

**Authors:** Inzamam Mashood Nasir, Muhammad Attique Khan, Mussarat Yasmin, Jamal Hussain Shah, Marcin Gabryel, Rafał Scherer, Robertas Damaševičius

**Affiliations:** 1 Department of Computer Science, HITEC University, Taxila 47080, Pakistan; inzamam.mashood@hitecuni.edu.pk (I.M.N.); attique.khan@hitecuni.edu.pk (M.A.K.); 2 Department of Computer Science, COMSATS University Islamabad, Wah Campus, Wah Cantonment 47040, Pakistan; mussaratabdullah@gmail.com (M.Y.); jamalhussainshah@gmail.com (J.H.S.); 3 Department of Intelligent Computer Systems, Częstochowa University of Technology, 42-200 Częstochowa, Poland; marcin.gabryel@pcz.pl (M.G.); rafal.scherer@pcz.pl (R.S.); 4 Faculty of Applied Mathematics, Silesian University of Technology, 44-100 Gliwice, Poland

**Keywords:** document classification, deep learning, feature selection, data augmentation, imbalanced dataset

## Abstract

Documents are stored in a digital form across several organizations. Printing this amount of data and placing it into folders instead of storing digitally is against the practical, economical, and ecological perspective. An efficient way of retrieving data from digitally stored documents is also required. This article presents a real-time supervised learning technique for document classification based on deep convolutional neural network (DCNN), which aims to reduce the impact of adverse document image issues such as signatures, marks, logo, and handwritten notes. The proposed technique’s major steps include data augmentation, feature extraction using pre-trained neural network models, feature fusion, and feature selection. We propose a novel data augmentation technique, which normalizes the imbalanced dataset using the secondary dataset RVL-CDIP. The DCNN features are extracted using the VGG19 and AlexNet networks. The extracted features are fused, and the fused feature vector is optimized by applying a Pearson correlation coefficient-based technique to select the optimized features while removing the redundant features. The proposed technique is tested on the Tobacco3482 dataset, which gives a classification accuracy of 93.1% using a cubic support vector machine classifier, proving the validity of the proposed technique.

## 1. Introduction

Document analysis and classification refer to automatically extracting the information and classifying it into a suitable category. Documents are often referred to as 2D material that can contain text or graphical items and can be used in optical character recognition (OCR) [1], word spotting [2], page segmentation [3], and cursive handwriting recognition [4] tasks. Document classification is considered as an essential step in classifying and analyzing the image documents. For several applications, classifying documents into their respective classes is a prerequisite step. If documents are well-sorted, it can be dispatched to the relative department for processing [5]. The indexing efficiency of a digital library can be improved with document classification [6]. Classifying the documents into content categories such as a table of content or a title page can suggest how pages extracting the metadata can be useful [7]. The retrieval efficiency and accuracy can be improved by classification on visual similarities, which can help users extract an article from any specific document or journal, containing a specific keyword, image, or table [8]. As the document classification is considered a higher-level analysis task, it is important to select the suitable document classes and types to get high accuracy and high performance in terms of effectiveness and efficiency [8].

Existing techniques either utilized the simple feedforward neural networks, standalone deep convolutional neural networks (DCNNs) models, or performed better on datasets, where a dataset contains a limited number of classes of documents. However, real-world cases have many issues in document classification, including structural similarities, low-quality images, and informational layers like signatures, marks, logo, and handwritten notes, which degrade the overall efficiency of many previously proposed methods. Data imbalance is also an essential problem in the deep learning (DL) domain, as overfitted and under fitted data can easily affect the overall performance of the proposed model. To resolve the problem of overfitting, the max-pooling layers have been added to the deep neural network models.

In this article, an automated system is proposed to classify the document images efficiently in accuracy and prediction time. We analyze and reduce the impact of adverse document image issues by employing multiple CNNs and combining each model’s training and properties. The selected primary dataset Tobacco3482 is hugely imbalanced, which is tackled by proposing a novel data augmentation technique. The secondary dataset RVL-CDIP is used to populate the minority classes. The fusion of multiple networks produces redundant features, which are tuned by employing the Pearson correlation coefficient (PCC)-based optimization technique.

The structure of this article is as follows. Details of the proposed technique are described in Section 3. Experimental results to validate the proposed technique are presented in Section 4, and Section 5 concludes this article with a conclusion and future directions in this research field.

## 2. Literature Review

Classification based on the content of document images has been broadly contemplated. Document classification can be performed using the visual-based local document image [9]. Structure models like letters and forms gave interesting results, when classified using region-based algorithms [10]. Morphological features such as text skew and handwriting skew have been addressed using entropy algorithm [11] and projection profiling [12]. The study of documents is commonly dependent on text removed using OCR techniques [13]. In another case, OCR is inclined to errors and is not generally pertain to every type of documents, e.g., the handwritten content is yet hard to peruse. A 4-layer Convolutional Neural Network (CNN) model was utilized for document classification using a small tobacco dataset for classifying tax forms [14]. This experiment outperformed the previous Horizontal-Vertical Partitioning and Random Forest (HVP-RF) and Speeded Up Robust Features (SURF) descriptor-based classification technique achieving an accuracy of 65%. Another technique for document classification utilizes principal component analysis (PCA) along with one-class support vector machine (OCSVM) in which PCA reduced the dimensionality and OCSVM performed the classification [15]. The PCA initially chose the top features for the document images from four different datasets. Then OCSVM was trained on selected features to classify the images into the most relevant classes with a precision rate of 99.62%. A semi-supervised learning approach utilizing CNNs based on graph-structured data was presented in [16]. The main idea is to localize the convolutions in an approximation of first-order spectral graphs. The model initially scaled according to the number of graph edges. It started learning the representations of hidden layers that encoded the features on the nodes and structure of local graphs. The approach was demonstrated on three datasets having 6, 7, and 3 classes, respectively.

In another work, multi-label document classification is applied to Czech newspaper documents, where features are extracted using a simple multi-layer perceptron and convolutional networks [17]. The achieved F1 score for this method was 84.0% while using a multi-layer perceptron with sigmoid functions. A biomedical document classification was carried out in [18], where an imbalanced bio-dataset was used for a cluster-based classification on the under-sampled dataset GXD. Overall precision of 0.72 was achieved. Another method involving a region-based training for document classification was proposed, which utilized the properties of the VGG16 model via transfer learning and achieved an accuracy of 92.2% on the Ryerson Vision Lab Complex Document Information Processing (RVL-CDIP) dataset [19].

The recent success of CNN [20] is inspired by novel deep learning applications such as breast cancer classification [21], fashion product classification [22], text sentiment analysis [23], computer network security [24], medical image analysis for disease diagnostics [25], speech recognition [26], semantic segmentation [27], malware classification [28], remote sensing [29], and document image analysis [30]. The CNN process is known as a supervised learning method, in which features are extracted and classified by a learning algorithm. Compositions are performed on the learned vectors for classification using deep learning methods. The performance of these networks is improved by collecting larger datasets, learning more powerful models, and avoiding overfitting using better techniques. These larger datasets include ImageNet [31], consisting of more than 15 million labeled images in 22,000 different categories, and LabelMe [32], consisting of millions of fully segmented images. The CNNs can learn from the larger datasets using different models [33]. These models’ capacity can be controlled by changing the order of layers to classify the input images correctly. As compared to the feedforward neural network with the same number of layers, CNNs contain fewer parameters and connections, making it easier and more convenient to test and train. As the use of graphical processing units (GPUs) has increased recently, many techniques have proposed effective and efficient ways to train CNNs using single and multiple GPUs [34]. After the success of a deep CNN model AlexNet [35], many more CNN models like GoogleNet [36], ZFNET [37], VGGNet [38], and ResNet [39] have also shown improved performance and results.

## 3. Proposed Method

**Figure 1:**
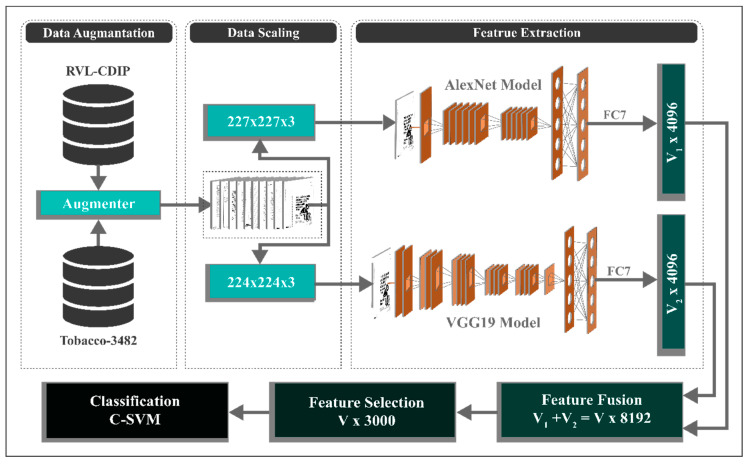
Detailed model of the proposed method.

For several applications, classifying documents into their respective classes is a prerequisite step. The indexing efficiency of a digital library can be enhanced with the help of document classification. There are numerous publicly accessible datasets for document classification, yet two acclaimed datasets, Tobacco3482 [40] and RVL-CDIP [41], are used, containing thousands of document images divided into 10 and 16 classes, respectively. These datasets have their challenges, and to get improved performance, a new technique utilizing the DCNN features is proposed having five significant steps, including (1) data balancing; (2) pre-processing; (3) feature extraction; (4) feature fusion, and (5) feature selection. In the first step, the imbalanced Tobacco3482 dataset is balanced using data augmentation technique. The dataset is then scaled down to the input sizes of both DCNN models and forwarded to pre-trained models, i.e., AlexNet and VGG19 to extract the DCNN features. Serial feature fusion is then applied on the DCNN features to fuse both models, which was finally optimized using the PCC-based technique [42]. These optimized features are forwarded to classifiers to obtain the classification accuracy. Additionally, a detailed model of the proposed technique is shown in Figure 1.

### 3.1. Data Augmentation

Imbalance of a dataset is a significant problem in any field as this can cause problems by ignoring the document images containing relevant information. Data imbalance occurs when one or more classes have a lower number of samples than the rest of the classes. Because of this problem, many well-modeled neural network architectures have failed to perform well. Imbalanced datasets in the domain of machine learning tend to produce unsatisfactory results. For any imbalanced dataset, if an event from minority class is predicted with an event rate of less than 5%, that is considered a rare event. The Logistic Regression and Decision Tree-based classification techniques tend to have a biased behavior toward rare events. These methods accurately predict the majority class, ignoring the minority class as noise. This eventually leaves a strong possibility of misclassifying the minority class when compared with the majority class.

This paper proposes a data augmentation-based approach to solve the data imbalance issue in an appropriate way. The following equations explain the process of solving this issue using the variables defined in Table 1.

The threshold T is defined as following, which represents the highest class of the dataset:

(1) $$T=\max\left(C_{i}\right),$$

where $C_{i}$ represents the sum of images in the *i*th class and i=1,…,n.

(2) $$D=\{\begin{array}{c} T-C_{i},if\;C_{i}<T\\ 0,\;if\;C_{i}\geq T \end{array},$$

where D is the difference between the threshold and the sum of a single class, which is computed by comparing $C_{i}$ with a threshold value. If D gives a non-zero value, it is forwarded to a function and the class label to fetch images from the secondary dataset to balance the primary dataset.

**Figure 2:**
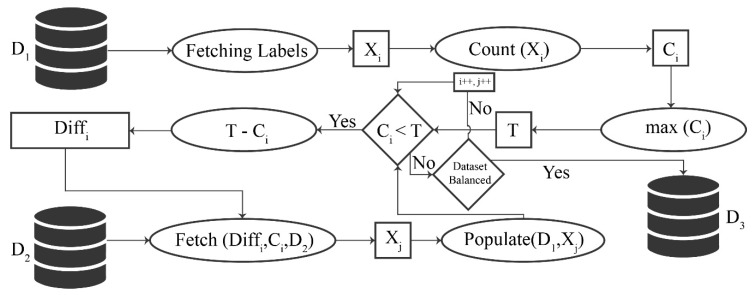
Flow diagram of data augmenter.

The flow diagram of the data augmenter is shown in Figure 2.

The algorithm for data balancing is mentioned below (see Algorithm 1). Here, the input is $D_{1\;}$ which denotes the Tobacco3482 dataset, while the output is $D_{3}$, which is an augmented, balanced dataset. Initially, all the labels are extracted from a dataset, which denotes all the classes. These labels are used to count images within each class, and a threshold value T is assigned with the highest-class count. The samples in all other classes are compared with T to calculate the difference. This difference, along with the class label and the secondary dataset $D_{2\;}$ is used to fetch the required number of images and populate the $D_{1\;}$ to form a new augmented dataset $D_{3}$.
**Algorithm 1.** Dataset balancing using a secondary dataset**Input:**$D_{1\;}$**Output:**$D_{3}$Step 1: $X_{i}\;\leftarrow D_{1}$Step 2: $C_{i}\;\leftarrow Count\left(X_{i}\right),\;where\;i=1,\ldots ,n$Step 3: $T\;\leftarrow \max\left(C_{i}\right)$Step 4: $Diff_{i}\;\leftarrow \;\{\begin{array}{c} T-C_{i},\;if\;C_{i}<T\\ 0\;,\;if\;C_{i}\geq T \end{array}$Step 5: $X_{j}\;\leftarrow Fetch\left(Diff_{i},C_{i},D_{2}\right)$ Step 6: $D_{3}=\;Populate\left(D_{1},X_{j}\right)$return $D_{3}$

The comparison of the primary dataset before and after augmentation is shown in Table 2. The classes in the primary and secondary datasets are also inserted in the table to make the comparison understandable. RVL-CDIP is a secondary dataset to balance the primary dataset (Tobacco3482). Table 2 shows the classes of both datasets. Left-most column present class names in a primary dataset, while the right-most column presents the corresponding classes from the RVL-CDIP dataset. The central columns present the number of images before and after data augmentation.

### 3.2. Network Architectures

Transfer of information between neurons is the primary motivation of CNNs. The CNNs have the same basic structure as classical artificial networks. The CNNs are composed of multiple layers which continuously fire neurons among connecting layers. The previous layer fires neurons onto the next layer as input, and each of these connections of successive layers is burdened with values called weights. The major difference between CNNs and classical networks is that classical networks accept the inputs in the form of vectors, while CNNs accept images as input data. The convolutional layer is the first layer of CNN, which receives an image from the input layer, and it uses an operation called image convolution to extract the features. To understand the functionality, a filter $f_{m,n}$ of size 3×3 is defined with a central position at m,n.

Many CNN models have pooling layers with each convolutional layer, which reduces the input image by selecting fewer pixels based on three major operations known as “max-pooling” “min-pooling”, and “average-pooling”. A pooling filter of size 3×3 will select only one value, which replaces all the nine values in the new vector representing the input image. The last layers of CNN models are always fully connected layers and separated into output layers or hidden layers. A tiny image described by numerical values is the input to these layers, which is already rectified by the previous combinations of convolutional and pooling layers. This layer uses an activation function to extract features from the rectified input image by creating multiple neurons and identifying the total units with each pixel value. The working of neurons can be described as:

(3) $$Out_{a}=\xi \left(\sum_{b}^{n}\omega_{a,b}In_{b}\right),$$

where $Out_{a}$ is an output of the current neuron, $In_{b}$ is input from the previous neuron, $\omega_{a,b}$ is the weight of the connection between *a*th and *b*th neuron and ξ is the activation function which is used to normalize the input values received from previous neurons to the range of (−1,1) can be further described as:

(4) ξ(In)=tanh(In),

#### 3.2.1. AlexNet

**Figure 3:**
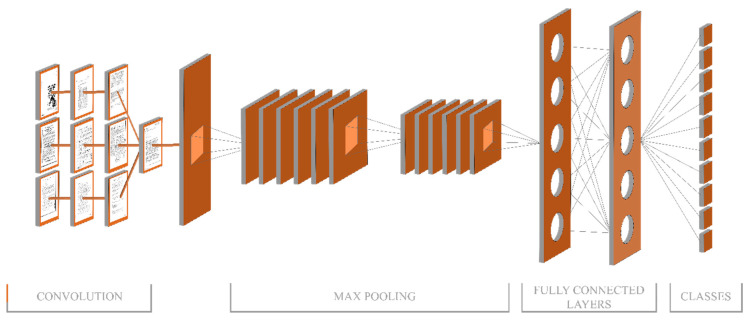
Structure of AlexNet Model.

The AlexNet has eight (8) distinguished layers, out of which five connected convolutional layers are at the beginning with pooling layers, followed by three (3) fully-connected layers. The output layer of this model is the softmax layer, which is directly connected with the last fully connected layer. The last layer is labeled as the FC8 layer, which fed the softmax layer with a feature vector of 1000 size, and softmax produces 1000 channels. Neurons of fully connected layers are directly attached to neurons of previous layers. Normalization layers relate to first and second layers. Fifth convolutional layer and response normalization layers have max-pooling layers. The output of every fully connected and convolutional layer has a ReLU layer. Input size for this network is 227×227×3. The AlexNet model structure used in this technique is shown in Figure 3 where FC7 is selected as an output layer.

#### 3.2.2. VGG19

**Figure 4:**
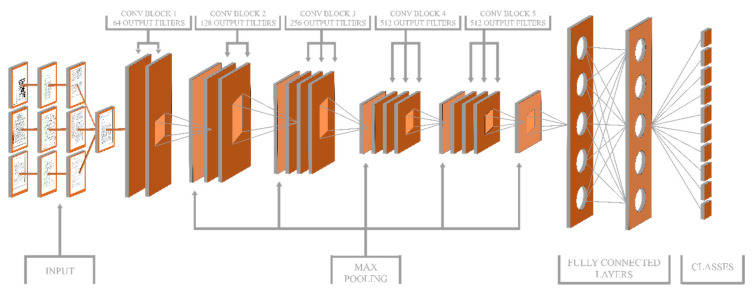
Structure of VGG19 Model.

Depth is an essential aspect of the CNN architecture. Increasing the layers of the network by adding more layers, a more significant CNN architecture was developed, which was more accurate on the ImageNet Large Scale Visual Recognition Challenge (ILSVRC) classification and localization tasks. The input to the VGG19 architecture is a fixed size RBG image of 224×224×3. Multiple convolutional layers accept the input image, which has the smallest sized 3×3 filters. The 1×1 convolutional filter was also used to transform the input channel from non-linearity to linear. One-pixel convolution stride is fixed, and the spatial resolution is fixed by the spatial padding for the convolutional layer. Five max-pooling layers carry the spatial pooling, out of which convolutional layers follow few. Having stride of 2, over a 2×2 pixel window, maximum pooling is applied. VGG19 also has three fully connected layers followed by a softmax layer at the end. The structure of the VGG19 model is explained in the following Figure 4, where FC7 is an output layer.

### 3.3. Feature Fusion and Selection

After extracting the deep features using two DCNN networks, AlexNet and VGG19, both features are serially fused to form a higher dimensional feature vector, which is explained as follows.

Suppose $a_{1},a_{2},a_{3},\ldots ,a_{n}$ belongs to a feature space $V_{1}$ and $b_{1},b_{2},b_{3},\ldots ,b_{n}$ belongs to the feature space $V_{2}$, and feature spaces $V_{1}$ and $V_{2}$ denote the DCNN features of AlexNet and VGG19, respectively. Feature spaces $V_{1}$ and $V_{2}$ are defined as:

(5) $$V_{1}=\left[\begin{array}{cccc} a_{1,1} & a_{1,2} & \cdots & a_{1,4096}\\ a_{2,1} & a_{2,2} & \cdots & a_{2,4094}\\ \vdots & \vdots & \vdots & \vdots \\ a_{n,1} & a_{n,2} & \cdots & a_{n,4096} \end{array}\right],$$

(6) $$V_{2}=\left[\begin{array}{cccc} b_{1,1} & b_{1,2} & \cdots & b_{1,4096}\\ b_{2,1} & b_{2,2} & \cdots & b_{2,4094}\\ \vdots & \vdots & \vdots & \vdots \\ b_{n,1} & b_{n,2} & \cdots & b_{n,4096} \end{array}\right],$$

(7) $$FV=V_{1}⊕V_{2},$$

(8) $$FV=\left[\begin{array}{cccccccc} a_{1,1} & a_{1,2} & \cdots & a_{1,4096} & b_{1,4097} & b_{1,4098} & \cdots & b_{1,8192}\\ a_{2,1} & a_{2,2} & \cdots & a_{2,4096} & b_{2,4097} & b_{2,4098} & \cdots & b_{2,8192}\\ \vdots & \vdots & \vdots & \vdots & \vdots & \vdots & \vdots & \vdots \\ a_{n,1} & a_{n,2} & \cdots & a_{n,4096} & b_{n,4097} & b_{n,4098} & \cdots & b_{n,8192} \end{array}\right],$$

where FV is a fused feature vector.

As both networks were trained to extract the features from fully connected layer FC7, a total of 4096 features were extracted and fused to form a new feature vector of size 8192 features. This fusion process compensates the inadequacy of a single network for document classification but increases the feature vector’s dimensions. Moreover, both networks use a basic CNN architecture with different approaches; there are chances of many correlations and redundant features among fused features.

Therefore, in this work, a PCC-based technique is implemented for selecting the optimized features by removing the redundant ones. The PCC-based feature selection technique evaluates different subsets of features based on highly correlated features [43].

The following equation explains the merit M of feature subset FV having i features:

(9) $$M_{FV_{i}}=\frac{i\times avg_{cf}}{\sqrt{i+i\left(i-1\right)avg_{ff}}},$$

where $avg_{cf}$ corresponds to the feature-classification correlations while $avg_{ff}$ corresponds to feature-feature correlations.

The criterion for the correlation coefficient-based feature selection CCFS can be defined as:

(10) $$CCFS=\underset{FV_{i}}{\max}\left[\frac{avg_{cf_{1}}+\;avg_{cf_{2}}+\ldots +avg_{cf_{i}}}{\sqrt{i+2(avg_{f_{1}f_{k}}+\ldots +avg_{f_{m}f_{n}}+\ldots +avg_{f_{i}f_{1}})}}\right],$$

where $avg_{cf_{i}}$ and $avg_{f_{m}f_{n}}$ are referred to as correlations between continuous features.

Suppose $W_{i}$ denotes the whole feature vector having $F_{i}$ features, then the equation mentioned above for CCFS can be rewritten as an optimized feature vector as:

(11) $$CCFS=\underset{w\in {\left\{0,1\right\}}^{i}}{\max}\left[\frac{{\left(\sum_{j=1,k=1}^{i}f_{i}w_{i}\right)}^{2}}{\sum_{j=1}^{i}w_{i}+\sum_{j\neq k}2\times f_{j}w_{j}w_{ij}}\right],$$

**Figure 5:**
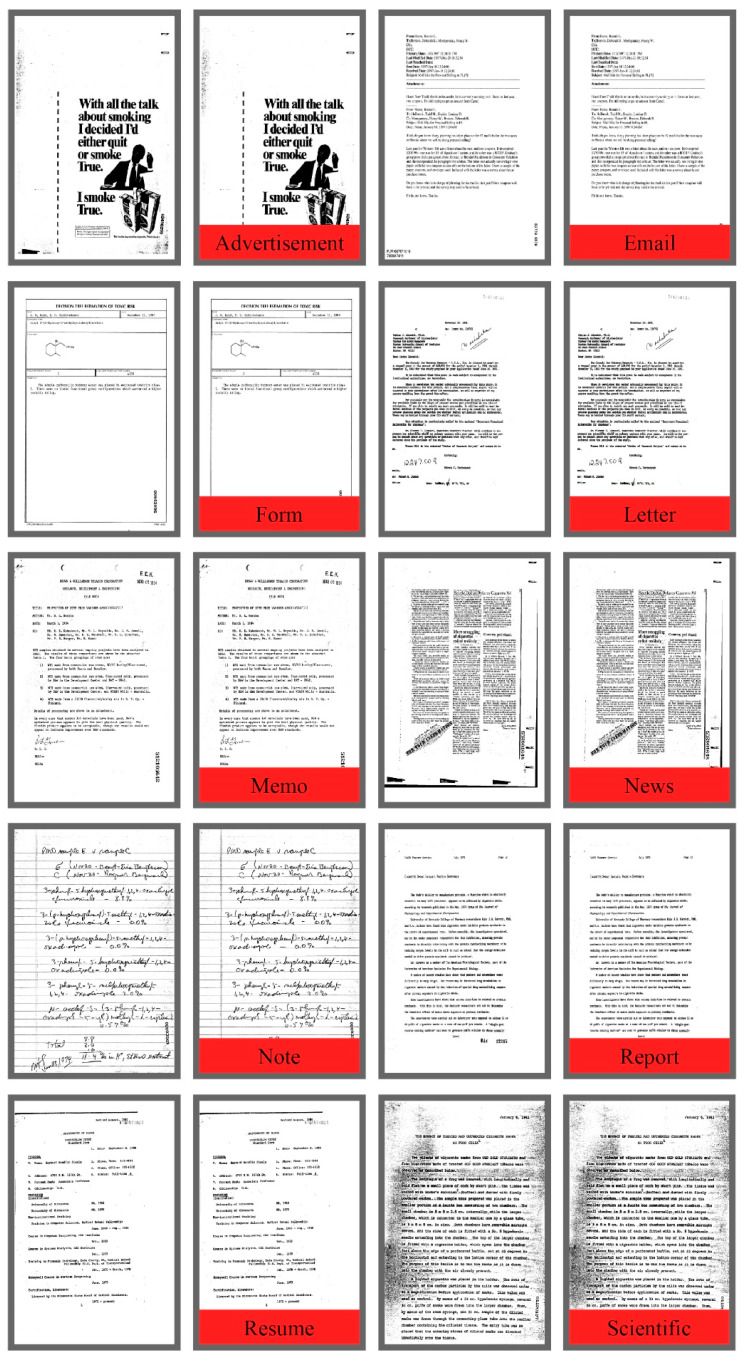
Labeled outputs of the proposed technique.

Features having a high correlation value are considered as redundant features, so only those features are selected, which have the minimum redundancy between consecutive features. The smallest Pearson’s correlation values concerning neighboring features are appended to the selected feature set. The feature vector’s final size becomes 3000 after selecting the best features and disregarding the redundant features. These best features are forwarded to the Cubic SVM (C-SVM) classifier to obtain the classification accuracy. The proposed technique is tested on the publicly available dataset Tobacco3482. The labeled outputs of the proposed technique are shown in Figure 5.

## 4. Experimental Results

### 4.1. Datasets

**Figure 6:**
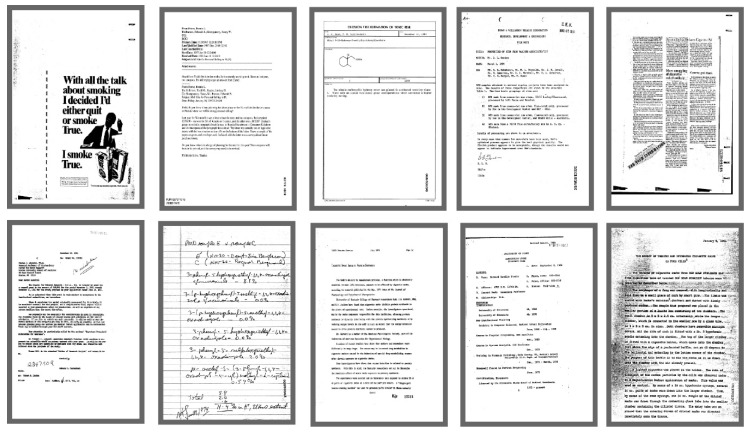
Sample images from Tobacco3482 dataset (one image per class). Left to right: (Advertisement, Email, From, Memo, News, Letter, Note, Report, Resume and Scientific).

The publicly available Tobacco3482 dataset is presented by a tobacco company including a different number of pictures per class, having 3482 pictures of high resolution from ten different classes. These images have a remarkable difference in structural and visual views, making this dataset more complex and challenging. The RVL-CDIP dataset is also a complicated, huge dataset that includes 400,000 labeled images in 16 different categories. In this article, RVL-CDIP was used as a secondary dataset for the augmentation purpose. The proposed technique is validated on the original Tobacco3482 dataset and an augmented dataset prepared during the data augmentation process. Few sample images from the Tobacco3482 dataset are shown in Figure 6.

### 4.2. Evaluation

The pre-trained DCNN models, i.e., AlexNet and VGG19, are used to extract the DCNN features by performing activations on the fully connected layer FC7. An approach of 50:50 split is adopted for training and testing to validate the proposed technique using ten-fold cross-validation. Ten machine learning methods (C-SVM, Linear Discriminant (LD), linear SVM (L-SVM), quadratic SVM (Q-SVM), fuzzy KNN (F-KNN), modified KNN (M-KNN), continuous KNN (C-KNN), weighted KNN (W-KNN), Subspace Discriminant, and Subspace KNN) were used as classifiers. All experiments are performed on Corei7, 7th generation with a 3.4 GHz processor, 16 GB RAM, 256 GB SSD having MATLAB 2018a (MathWorks Inc., Natick, MA, USA).

### 4.3. Classification Results

Three experiments are performed to obtain classification results such as (a) classification using the AlexNet features with PCC-based optimization; (b) classification using VGG19 features with the PCC-based optimization; (c) classification using a fusion of AlexNet and VGG19 features with the PCC-based optimization. Classification accuracy and execution time are validated by comparing it with the state-of-the-art techniques applied to the same dataset and sub-dataset.

**Figure 7:**
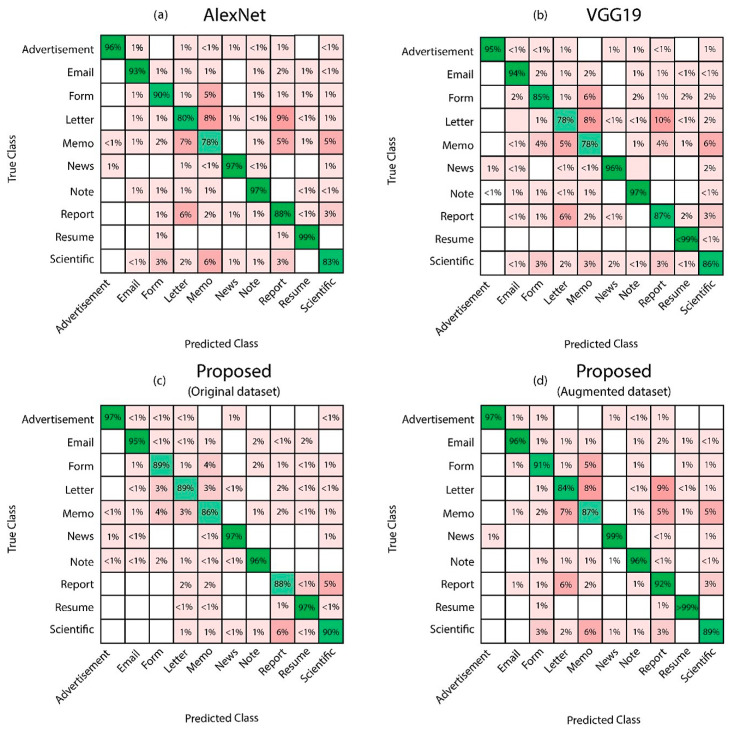
Confusion matrices for the Tobacco3482 dataset: (**a**) AlexNet, (**b**) VGG19, (**c**) proposed method on the original dataset, and (**d**) proposed method on the augmented dataset.

AlexNet DCNN with PCC-based Optimization: In the first experiment, the AlexNet model is used to extract DCNN features that are reduced using the PCC-based optimization to select the best features. Selected 3000 features were then forwarded to ten (10) different classifiers. The best classification accuracy of 90.1% and false-negative rate (FNR) of 9.9% is achieved using C-SVM with a training time of 670.8 s. The confusion matrix, shown in Figure 7a, confirms the accuracy of C-SVM. Q-SVM achieves the second-best accuracy with 89.6% and FNR of 10.4% in execution time of 742.2 s. Overall results of this experiment on different classifiers is displayed in Table 3.

VGG19 DCNN with PCC-based Optimization: In this experiment, VGG19 is used for DCNN feature extraction and PCC selected the optimized features. Selected 3000 features are then forwarded to ten (10) different classifiers, out of which, the best classification accuracy at 89.6% and FNR of 10.4% is recorded on C-SVM with a training time of 947.3 s. The classification accuracy of Cubic SVM is confirmed by the confusion matrix shown in Figure 7b. The second highest accuracy of 87.1% with FNR of 12.9%, and training time of 1996 s was achieved on Q-SVM. The detailed results of this experiment on multiple classifiers are listed in Table 3 as well.

AlexNet and VGG19 DCNN feature fusion and PCC-based Optimization: A serial-based fusion approach is applied to fuse the DCNN features of AlexNet and VGG19 models, which are later optimized using the PCC-based selection. Both DCNN models extracted 4096 features each, and feature fusion strategy is applied to combine both models’ characteristics.

The proposed technique is validated on two cases for a fair comparison with existing techniques. Initially, the proposed technique is validated using the original imbalanced Tobacco3482 dataset, where it achieved the highest accuracy of 92.2% with FNR of 7.8% and training time of 329.5 s on C-SVM classifier. While in another case, it is validated using an augmented dataset after the augmentation process described in the proposed section, where the original dataset was balanced using a secondary dataset RVL-CDIP. C-SVM achieved the best accuracy of 93.1% in 364.1 s with FNR of 6.9%. Figure 7c,d shows the confusion matrices, which confirms classification accuracy of Cubic SVM on both cases. Table 4 contains the results of all experiments mentioned above on ten selected classifiers along with respective accuracies, FNR, and training time. There are other experiments, which are carried out to validate the proposed model. Table 4 illustrates the results after feature fusion. The highest accuracy of 91.5% is achieved using C-SVM. It is noteworthy that this experiment’s training time increases as the total number of features increased after fusion. The fusion increases the chances of redundant and irrelevant features, which are removed by employing PCC-based feature selection technique.

### 4.4. Discussion

We discuss the significance of proposed results on several classifiers. Without statistical analysis, it is not clear that which classifier outperforms for document classification. Therefore, we have conducted more experiments and computed standard deviation, confidence interval (CI), denoted by $\sigma_{\bar{x}}$ and margin of error at confidence level (95%, 1.96$\;\sigma_{\bar{x}}$). The values are tabulated in Table 5 and Table 6. In Table 5, the minimum accuracy achieved on C-SVM after 100 iterations is 90.7% whereas the average and best accuracies are 91.45% and 92.2%, respectively. The value of σ is 0.75 and $\sigma_{\bar{x}}$ is 0.5303, respectively. The margin of error on confidence level (CL) (95%, 1.96$\;\sigma_{\bar{x}}$) is 91.45 ± 1.039 (±1.14%), which is better as compared to other classifiers. Similarly, the analysis is also conducted on the augmented dataset and values are tabulated in Table 6. For C-SVM, CL (95%, 1.96$\;\sigma_{\bar{x}}$) is 92.7 ± 0.554 (±0.60%), which is better as compared to other classifiers performance.

Several previous techniques had also used the Tobacco3482 dataset to validate their models. A custom CNN-based architecture, inspired by AlexNet, was proposed in [44] for document classification. Multiple experiments were performed including 20 images per class and 100 images per class for training and validation, respectively, and achieved classification accuracies of 68.25% and 77.6%, for both tests respectively. Another approach utilized DCNN model as a feature extractor and extreme learning machine (ELM) for classification in [45]. Overall accuracy of 83.24% was achieved on the Tobacco3482 dataset. A DCNN-based approach utilizing AlexNet, VGG16, GoogLeNet, and ResNet-50 was proposed in [46], where classification accuracy of 91.13% is recorded. In [47], a spatial pyramid model is proposed to extract high discriminant multi-scale features of document images by utilizing the inherited layouts of images. A deep multi-column CNN model is used to classify the images with an overall classification accuracy of 82.78%. In [48], combining semantic information with visual information of images allowed an improved separation toward document classification. The model has tested on the Tobacco800 [49] dataset and achieved an accuracy of 93%. Tobacco-800 is a subset of the actual Tobacco3482 dataset, with fewer classes. The purpose of comparing this dataset is to validate the proposed methodology demonstrating that it still outperforms other techniques tested with less classes. The performance of related work is summarized in Table 7.

The proposed technique obtained a classification accuracy of 93.1% with an average training time of 364.17 s and an average prediction time of 0.78 s. Note that the proposed technique’s training time increases when it is tested on the augmented dataset due to the increased number of images in each class. But as the training proceeds, the prediction time is reduced in half, which shows the balanced dataset’s importance.

## 5. Conclusions

In this article, a hybrid approach to classify the documents using deep convolutional neural networks is proposed, consisting of data augmentation, data normalization, feature extraction, feature fusion, and feature selection steps. In the data augmentation step, the dataset is analyzed, and classes within the dataset with fewer images are fed using the secondary dataset RVL-CDIP. After that, data normalization is performed, which resized the dataset images according to pre-trained models’ sizes. The pre-trained AlexNet and VGG19 models are used to extract deep features, which are fused using a serial-based fusion, and, in the end, the Pearson correlation coefficient-based technique selects the best features. The selected features are then forwarded to the Cubic SVM classifier for document classification. The proposed technique is validated on the publicly available Tobacco3482 dataset, achieving an accuracy of 93.1%. The obtained results outperformed the previous techniques and validated the proposed technique.

Moreover, this technique reduces training and prediction time, which is also an essential development in the document classification field. There are several open questions for this research including: (a) The selection of CNN models (other pre-trained or custom CNN models may perform better on this domain); (b) the selection of the technique to fuse different features is also not a limitation, as there are several other fusion techniques [50,51,52,53], which can perform better; and (c) feature selection technique used in this work is also not a limitation as other feature selection methods can also be implemented and tested.

In the future, a new generic method for document image classification will be developed by combining the hand-crafted features with the DCNN features to achieve a further improved classification accuracy. Furthermore, a real-time application also will be developed to classify documents in real-time.

## Figures and Tables

**Table 1:** Nomenclature of variables used in the definitions and equations.

Variable	Description	Variable	Description
T	Threshold Value	$C_{i}$	Sum of images in the *i*th class
D	Difference between the threshold and the sum of a single class		
$D_{1}$	Tobacco3482 dataset	$D_{2}$	RVL-CDIP dataset
$D_{3}$	Balanced Dataset	$In_{b}$	Input from the previous neuron
$Out_{a}$	Output of the current neuron	$\omega_{a,b}$	Weight of the connection between *a*th and *b*th neuron
ξ	Activation function	$V_{1}$	DCNN features of AlexNet
$V_{2}$	DCNN features of VGG19	$M_{FV_{i}}$	The merit M of feature subset FV having i features
$avg_{cf}$	Feature-classification correlations	$avg_{ff}$	Feature-feature correlations
$F_{i}$	*i*th feature	$W_{i}$	*i*th weight

**Table 2:** Dataset before and after applying the data augmentation algorithm.

Classes in Tobacco3482	# of Images before Augmentation	# of Images after Augmentation	Classes in RVL-CDIP
Advertisement	230	620	Advertisement
Email	599	620	Email
Form	431	620	Form
Letter	567	620	Letter
Memo	620	620	Memo
News	188	620	News Article
Note	201	620	Handwritten
Report	265	620	Scientific Report
Resume	180	620	Resume
Scientific	261	620	Scientific Publication

**Table 3:** Comparison of classification accuracy, false-negative rate (FNR), and Training Time on Tobacco3482 Dataset. Best values are shown in bold.

Method	Experiments				Performance Measures		
	AlexNet	VGG-19	Proposed (Original Dataset)	Proposed (Augmented Dataset)	Accuracy (%)	FNR (%)	Training Time (s)
**C-SVM**	√				**90.1**	**9.0**	**670.8**
		√			**89.6**	**10.4**	**947.3**
			√		**92.2**	**7.8**	**329.5**
				√	**93.1**	**6.9**	**364.1**
Linear Discriminant	√				79.7	20.3	772.5
		√			-	-	-
			√		82.4	17.6	593.2
				√	84.0	16.0	659.9
L-SVM	√				81.6	18.4	1731.7
		√			79.0	21.0	2198.9
			√		81.8	18.2	971.3
				√	84.3	15.7	1170.2
Q-SVM	√				89.6	10.4	742.2
		√			87.1	12.9	1996.0
			√		87.4	12.6	582.6
				√	91.8	8.2	625.2
F-KNN	√				87.1	12.9	846.8
		√			83.7	16.3	1720.9
			√		85.0	15.0	742.6
				√	89.5	10.5	872.9
M-KNN	√				73.8	26.2	744.9
		√			65.5	34.5	1920.3
			√		73.9	26.1	621.1
				√	76.5	23.5	767.4
C-KNN	√				74.0	26.0	1604.8
		√			65.9	34.1	4147.5
			√		72.8	27.2	598.4
				√	76.4	23.6	719.1
W-KNN	√				87.1	12.9	951.4
		√			83.0	17.0	2393.5
			√		84.3	15.7	687.2
				√	88.7	11.3	746.9
Subspace Discriminant	√				89.5	10.3	5305.0
		√			87.5	12.5	6304.3
			√		88.3	11.7	1716.8
				√	89.7	10.3	2079.2
Subspace KNN	√				87.0	13.0	2498.6
		√			83.2	16.8	2508.9
			√		86.9	13.1	1958.2
				√	89.4	10.6	2398.9

**Table 4:** Classification results after feature fusion. Best values are shown in bold.

Classifier	Performance Measures					
	Sensitivity (%)	Precision (%)	AuC (%)	FNR (%)	Accuracy (%)	Training Time (s)
C-SVM	**91.6**	**91.6**	**99.3**	**8.50**	**91.5**	3037.7
Linear Discriminant	81.2	81.3	89.7	18.7	81.3	3055.7
L-SVM	84.5	84.8	98.1	15.6	84.4	2861.1
Q-SVM	90.3	90.3	99.0	9.70	90.3	2989.4
F-KNN	86.7	87.0	92.6	13.2	86.8	2176.9
M-KNN	75.0	76.3	95.5	24.9	75.1	2176.7
C-KNN	74.5	76.0	95.3	25.5	74.5	5307.6
W-KNN	86.9	87.4	98.5	13.0	87.0	**2174.4**
Subspace Discriminant	86.1	86.2	98.5	13.7	86.3	8794.6
Subspace KNN	86.9	87.0	95.5	13.0	87.0	3876.5

**Table 5:** Analysis of proposed method on original data. Best values are shown in bold.

Method	Min (%)	Avg (%)	Max (%)	σ	$\sigma_{\bar{x}}$	$\mathrm{ME}\;(95\%,\;1.96\;\sigma_{\bar{x}})$
**C-SVM**	**90.7**	**91.45**	**92.2**	**0.75**	**0.5303**	**91.45 ± 1.039 (±1.14%)**
LD	79.4	80.90	82.4	1.5	1.0606	80.9 ± 2.079 (±2.57%)
L-SVM	78.3	80.05	81.8	1.75	1.2374	80.05 ± 2.425 (±3.03%)
Q-SVM	84.8	86.10	87.4	1.3	0.9192	86.1 ± 1.802 (±2.09%)
F-KNN	83.2	84.10	85.0	0.9	0.6363	84.1 ± 1.247 (±1.48%)
M-KNN	70.6	72.25	73.9	1.65	1.6670	72.25 ± 2.87 (±3.17%)
C-KNN	71.1	71.95	72.8	0.85	0.6010	71.95 ± 1.178 (±1.64%)
W-KNN	81.6	82.95	84.3	1.35	0.9545	82.95 ± 1.871 (±2.26%)
ESDA	85.4	86.85	88.3	1.45	1.0253	86.85 ± 2.010 (±2.31%)
ESKNN	83.2	85.05	86.9	1.85	1.3081	85.05 ± 2.564 (±3.01%)

**Table 6:** Analysis of proposed method on augmented dataset. Best values are shown in bold.

Method	Min (%)	Avg (%)	Max (%)	σ	$\sigma_{\bar{x}}$	$\mathrm{ME}\;(95\%,\;1.96\;\sigma_{\bar{x}})$
**C-SVM**	**92.3**	**92.7**	**93.1**	**0.4**	**0.2828**	**92.7 ± 0.554 (±0.60%)**
LD	81.7	82.8	84.0	1.15	0.8131	82.85 ± 1.594 (±1.92%)
L-SVM	82.6	83.4	84.3	0.85	0.6010	83.45 ± 1.178 (±1.41%)
Q-SVM	89.4	90.6	91.8	1.2	0.8485	90.6 ± 1.663 (±1.84%)
F-KNN	87.1	88.3	89.5	1.2	0.8485	88.3 ± 1.663 (±1.88%)
M-KNN	73.8	75.1	76.5	1.35	0.9545	75.1 ± 1.871 (±2.59%)
C-KNN	73.6	75.1	76.4	1.4	0.9899	75.0 ± 1.940 (±2.59%)
W-KNN	84.9	86.8	88.7	1.9	1.3435	86.8 ± 2.633 (±3.03%)
ESDA	85.7	87.7	89.7	2.0	1.4142	87.7 ± 2.772 (±3.16%)
ESKNN	86.3	87.8	89.4	1.55	1.0969	87.85 ± 2.148 (±2.45%)

**Table 7:** Comparison with existing techniques on the Tobacco3482 dataset.

Paper	Dataset	Accuracy (%)	Training Time (s)	Prediction Time (s)
Afzal et al. [44]	Tobacco3482	77.6	-	-
Kölsch et al. [45]	Tobacco3482	83.24	-	-
Afzal et al. [46]	Tobacco3482	91.13	-	-
Sarkhel & Nandi [47]	Tobacco3482	82.78	-	-
Wiedemann & Heyer [48]	Tobacco-800	93	-	-
Proposed	Primary: Tobacco3482 Secondary: RVL-CDIP	AlexNet: 90.1	670.8	2.34
		VGG19: 89.6	947.3	3.95
		Original: 92.2	329.5	1.62
		Augmented: 93.1	364.1	0.78
